# A Clinical Demonstration

**Published:** 1896-12-12

**Authors:** Pearce Gould

**Affiliations:** Surgeon, Middlesex Hospital; and Teacher of Practical and Operative Surgery, Middlesex Hospital Medical School.


### A CLINICAL DEMONSTRATION.

By Mr. Pearce Gould, M.S., F.R.C.S., Surgeon, Middlesex Hospital; and Teacher of Practical and Operative Surgery, Middlesex Hospital Medical School.

Mr. Pearce Gould: The history of this case is as follows: The man is thirty years of age, a coal miner by occupation. In July, 1894, *i.e.*, two years and four months ago, he was working in a coal mine, bending forwards, when a large mass of coal fell on his back, striking him between the shoulders. He was thrown forwards, but did not become unconscious. When examined, it was found that he had lost sensation and motion in both his lower extremities, and in this condition he has remained ever since. We want to find out to-day what the condition of his lower limbs and spine is, and then to talk about what we can do to relieve him. Taking the condition of his lower limbs first, what points must we investigate?

Student: We can see that he has lost the power of movement in his legs. We want, therefore, to find out whether the patient has lost all or any of the different kinds of sensation, and the extent of the area that is anæsthetic and paralysed.

Mr. P. G.: What else? Assuming for a moment—not a groundless assumption by any means—that he has injured the contents of his spinal canal, what other conditions might we expect?

S.: We might look for atrophy of the muscles, insensibility to the application of electricity, and the absence of reflex action.

Mr. P. G.: Very well. We have to look, therefore, for changes in the sensations, in the reflexes, in the nutrition of the limbs, and in the electrical reactions. What shall we take first?

S.: The sensations, *i.e.*, the sensibility to touch, to pain, to heat, and to cold.

Mr. P. G.: What would you use in order to test the first of these sensations?

S.: What you would use should not convey a sense of heat or cold, or weight, or pain. I should think the feather end of a quill.

[The result of examination was that the patient was found to have lost sensation of touch up to just above the groin.]

Mr. P. G.: Now another form of sensation?

S.: Heat or cold.

Mr. P. G.: Are they the same?

S.: No. Two different sets of nerves are said to be involved in the perception of these conditions.

Mr. P. G.: How will you test?

S.: With test tubes, filled in the one case with hot water, and in the other with iced water.

Mr. P. G.: To what part of the limbs will you apply your tests? Is there any one part of the lower limb which is more sensitive to heat and cold than another?

S.: The inner side of the knee.

Mr. P. G.: In the application of the heat test in cases of paralysis you must remember to use great care, for if there is paralysis of the nutritive nerves, you may produce a blister with water that is only comfortably warm to your own hand.

[On applying this test the patient was found to be insensitive to heat and cold over the anæsthetic area.]

Mr. P. G.: Now what other sensation have we to test?

S.: The sensation of pain.

Mr. P. G.: How would you test that?

S.: With the point of a quill pen.

Mr. P. G.: Why not with a pin or a needle?

S.: You might start a sore.

Mr. P. G.: You mean to say that you might infect the man. Well, that is true. But there is another reason, too. It is quite possible to put the point of a pin or of a needle into the limb without its coming into contact with any of the pain-conducting nerves. Therefore, too sharp a point is not wanted, and that of a quill pen fulfils the conditions as nearly as possible.

[On applying this test the area of absence of sense of pain was found to correspond with that of loss of tactile sensation.]

Mr. P. G.: What we have therefore found out is that the patient has lost all sensation over the part mapped out. He has no tactile sensation, no sensation of pain, nor of heat, nor of cold. We have now to test his muscular sense. What is the muscular sense?

S.: Sense of resistance to muscular contraction or tension upon the muscle, and the sensation that enables us to judge of the exact position of our limbs.

The patient's eyes being shut, his legs were placed across one another, and he was asked—

S.: Can you tell me in what position your legs are now?

Patient: No.

Mr. P. G.: Try the other leg. Can you tell now?

P.: No.

Mr. P. G.: You see he has also entirely lost the muscular sense. Now see if he is paralysed.

S. (to patient): Can you move your legs at all?

P.: No.

S.: Can you bend your knee?

P.: No.

Mr. P. G. (to student): Can you yourself apply any test? If you lift up a paralysed limb, what happens?

S.: It drops down. Then, too, in a paralysed leg the toes offer no resistance and feel perfectly limp, and the leg itself tends to roll over towards its outer side. All these signs are present in this case.

Mr. P. G.: Then we have arrived at these facts: The patient is in the position of a paralysed man; if his limbs are lifted up they drop down at once; the toes offer no resistance; and if you ask the man to exercise his will he does not make any muscular contraction at all. Now, we want to find out the extent of the paralysis. Ask him questions with this object.

S. (to patient): Can you raise yourself in bed?

P. (doing so with the assistance of his arms): Yes.

Mr. P. G.: What muscles did he use for that?

S.: His abdominal muscles and the muscles of his arms, for he half lifted himself up by them.

Mr. P. G.: We will now ask him to try to raise himself without using his arms. You see he merely bends his spine, but cannot move the pelvis on the thighs—cannot flex the hip-joint. What does that tell us?

S.: That the flexors of the hip are paralysed.

Mr. P. G.: What are they?

S.: Principally the psoas and iliacus.

Mr. P. G.: Yes. We find, then, that the paralysis extends up as high as the psoas and iliacus, but does not involve the abdominal muscles. The abdominal muscles are supplied by the lower dorsal nerves, the psoas and iliacus by the second and third lumbar nerves.

Mr. P. G.: That clears up the question as to the level of the motor paralysis. Let us try the reflexes.

[The reflexes in the lower limbs were absent, but in the abdomen were well marked.]

Mr. P. G.: He has lost the skin reflexes and the deep tendon reflexes. What else do we want to observe?

S.: We want to note any trophic changes.

Mr. P. G.: What are they?

S.: Wasting of the muscles, stiffness of the joints, and atrophic changes of the skin.

Mr. P. G.: Yes; taking the last first, do you notice anything about the skin?

S.: It is flabby to the touch, and is of a lighter colour than that of the rest of the body.

Mr. P. G.: Quite so. There is a very marked difference in the pigmentation.

S.: The nails look brittle.

Mr. P. G.: As they were cut to-day we cannot say much about their rate of growth, but the nurse says they were very thick. The most marked change in the skin is the absence of pigmentation. Then, too, there is a very marked wasting away of the muscles. What other trophic changes would you look for?

S.: Stiffness of the joints and lowered temperature. There is a very marked stiffness of the knees and ankles, but none in the hip. The temperature of the paralysed limbs is distinctly colder than the rest of the body.

Mr. P. G.: What changes have taken place in the joints, and how is it that the hip joint is not affected?

S.: There is a thickening in the cartilages, followed by erosion, and ultimately adhesions form between the surfaces.

Mr. P. G.: The movement in the knee is so smooth that I do not think we have splitting up of the cartilage, but the ligaments have thickened, hardened, and lost their suppleness and elasticity. Possibly there are adhesions, but I do not think this is so. The hips do not show these changes, probably because the man has been able to move these joints by the help of his arms, but the knees and ankles have been motionless for a very long time.

[On testing the electrical reaction there was no response to either form of current.]

[The patient was then turned over on his face, and the region of anæsthesia was marked out, when it was found to extend up to the iliac crest on each side, except over the part of skin of the right buttock supplied by the last dorsal nerve.]

Mr. P. G.: Now we want to examine the spine. We find that there is a prominence in the upper lumbar spine, one of the vertebræ being pushed quite over to the right side, and, in addition, there is a considerable thickening of the lumbar spines. The displaced spine is that of the first lumbar vertebra, and the last dorsal and the first three lumbar spines are involved in a thickened, prominent mass of bone.

[The patient was then turned over on to his back.]

Mr. P. G.: Now we have to examine his bladder. What do we want to learn?

S.: Whether he passes his water involuntarily.

Mr. P. G. (to patient): Are you able to make water when you want to?

P.: Yes.

S.: Do you know when you want to make water?

P.: No.

S.: In what way can you pass it off?

P.: By bringing a force to bear down in that direction.

Mr. P. G.: When he contracts his abdominal muscles, he means. When do you force down?

P.: When I want to clear my bladder before going out.

Mr. P. G.: Can you feel that you want to clear it?

P.: No.

Mr. P. G.: He does not do this as the result of any sensation, not because he has any desire to pass water, but because he has learnt that he has the power by squeezing the bladder to empty it, and thereby prevent its involuntary emptying at the wrong time. It does not mean that he has any control over his bladder. What condition do we expect to find in the bladder in the case of an injury to the spine?

S.: Either continence or incontinence.

Mr. P. G.: Explain what you mean.

S.: Either the urine runs out of the urethra as fast as it runs into the bladder, the bladder having no power of retaining it—that is incontinence; or the urine runs into the bladder, accumulates there, and after a time is discharged—continence.

Mr. P. G.: In the latter case we have a very feeble imitation of the natural process. (To patient): How often do you empty your bladder?

P.: Sometimes about every two hours, but at other times oftener.

The catheter was then passed, with the result that 3½ oz. of urine was drawn off.

It was also elicited that the patient had no power over his rectum, no sensation on defæcation, but there was a distinct rectal reflex when the finger was pressed into the bowel.

Mr. P. G.: Does this condition of things correspond at all with the condition of the lower limbs? Perhaps, however, I had better ask something else first. Where does the spinal cord end?

S.: Opposite the cartilage between the first and second lumbar vertebræ.

Mr. P. G.: Where is the lumbar enlargement of the cord?

S.: Opposite the twelfth dorsal and the first lumbar vertebræ.

[Here the position of these parts was marked on the skin.]

Mr. P. G.: Now, does this deformity correspond with the symptoms in the lower part of the body?

S.: Pretty nearly in everything.

Mr. P. G.: What nerves supply sensation to the abdominal wall?

S.: The dorsal nerves.

Mr. P. G.: And what to the extremities?

S.: The lumbar and sacral nerves.

Mr. P. G.: Roughly, it may be said that the groin marks the break between the skin supply arising from the dorsal and the lumbar nerves. If the injury took place just where the first lumbar nerve comes out below the first lumbar vertebra, we should have entire anæsthesia of the lower limb except over a little patch—as we have in this case—just below the ilium, which has sensation supplied by the twelfth dorsal nerve. The lower dorsal nerves supply the abdominal muscles. Therefore, if these nerves have escaped injury, the abdominal muscles would act as they do in this case.

Mr. P. G.: Now to sum up the result of our examination. This man has complete motor and sensory palsy of his lower limbs, the exact limit of the palsy being between the twelfth dorsal and second lumbar nerves. All the reflexes in the limbs are lost, but he retains his bladder and his rectal reflex. The paralysed muscles have lost all reaction to galvanism, showing that not only is there interruption of the nervous path, but that the muscular tissue itself has disappeared. This palsy is the result of an injury to the spine, and the deformity he now presents suggests that the maximum intensity of that injury was at the level of the first lumbar vertebra. We cannot doubt that the contents of the spinal canal were crushed by the fractured spine. It is an exceedingly difficult thing in many cases to determine whether the cauda equina or the spinal cord itself is crushed; when the seat of the fracture affords no certain guide, the completeness or incompleteness of the paralysis is the point to rely upon. The spinal cord is so soft and easily lacerated that compression by a displaced vertebra is almost certain to crush it entirely and destroy all its functions. The nerves of the cauda equina, however, are tougher—more resistant—and so it happens that when they are compressed by a displaced bone some one or more nervous path is preserved, and the paralysis is not so complete.

Applying that test to this case, we find that there are two nerve-paths preserved below the level of general paralysis—those for the rectal and the bladder reflexes, and that the centres of these reflexes in the grey matter of the lumbar enlargement of the cord are also intact. From this I infer that the lumbar enlargement of the cord is not the seat of a paralysing crush, but that the fracture has involved the nerves of the cauda equina, and has exerted such pressure as to interrupt all the nervous paths except those of these two visceral reflexes —a very interesting condition.

The man has come into the hospital in the hope that laminectomy might afford him some relief. Unquestionably that operation was indicated immediately after the accident, and might then have been followed by great benefit. But I do not think the operation would be justifiable now. In the first place, after this lapse of time—nearly two and a half years—we could not hope for any repair in these injured nerves. But the entire loss of all response of the muscles to galvanism shows that, even were the paths of nervous impulses restored, the muscles would not be regenerated, and he would still remain a paralysed man.

